# The Physician’s Visiting List for 1868

**Published:** 1867-10

**Authors:** 


The Physician's Visiting List for 1868. Philadelphia: LINDSAY & BLAKISTON.

This pioneer work of its kind, and still one of the most convenient for the daily use of the practioner, is already issued in form for 1868, and may be found on the tables of all our medical booksellers.

